# Limitations of Risk and Symptom-Based Screening: Closing the Gap

**DOI:** 10.1016/j.jacadv.2026.102701

**Published:** 2026-04-04

**Authors:** Richard A. Chazal

**Affiliations:** Lee Health Heart Institute, Fort Myers, Florida, USA

A recent report described shortcomings of current risk assessment to predict a type I myocardial infarction.^1^ Of the patients, 45% to 61% presenting with first MI would not have been candidates for guideline-based statins or further imaging if seen 2 days before the event.

The authors note that the volume of people at risk for ischemic events results in a substantial number of individuals who suffer harm using the current population-based prevention approach. They further point out rapidly evolving evidence connecting plaque burden and type with subsequent events.

Some tools are available currently. Plaque can be detected by coronary calcification with the caveat that many patients have only noncalcified plaque (particularly younger individuals). Computed cardiac tomographic angiography (CCTA) closes some detection gaps but involves a more complicated process and higher costs. Nevertheless, CCTA (and possible use of quantitative methodologies) holds promise in migrating toward personalized care.^2^ One randomized trial (TRANSFORM^3^) and one registry (DECIDE^4^) are currently exploring this premise.

TRANSFORM and DECIDE will hopefully inform future practice. In the interim, clinicians can mine available data to improve prediction (and treatment) of risk. Coronary calcium scoring is inexpensive and is currently recommended in guidelines for some intermediate risk patients. Attention to incidentally discovered vascular calcification on nongated computed tomography scans is important, with artificial intelligence tools for quantitating incidentally discovered calcification commercially available. Compliance with chest pain guidelines can result in an increasing number of persons evaluated for chest discomfort with CCTA. This yields increased detection of plaque, with the aspiration of effective prevention.^3^

Cardiovascular clinicians desire definitive data to alter practice but substantial evidence, including this report,^1^ reminds us of our duty to use the best available information toward the goal of improving heart health for all. The time is on us for prevention paradigms to evolve.
